# Elucidating the role of peripheral monocyte nicotinic acetylcholine receptors and inflammation in cognitive outcomes in older adults

**DOI:** 10.1007/s10522-025-10220-3

**Published:** 2025-03-30

**Authors:** Jordan N. Kohn, Gavrila Ang, Christopher Pruitt, Isabel Gandarilla, Xin Tu, Suzi Hong

**Affiliations:** 1 Herbert Wertheim School of Public Health and Human Longevity Science, University of California San Diego, 9500 Gilman Dr., #0804, La Jolla, CA 92093 USA; 2 Department of Psychiatry, University of California San Diego, La Jolla, CA 92093 USA

**Keywords:** Immune aging, Cognition, Monocytes, Acetylcholine receptors, Anticholinergic burden

## Abstract

Nicotinic acetylcholine receptors (nAChRs) are important regulators of brain and immune function that play critical roles in the neuropathology and progression of Alzheimer’s disease and related dementias (ADRD). However, quantifying nAChRs in the brain remains elusive, and little is known about peripheral measures of nAChR in older adults or their relationship to cognition. Here, we examined associations between nAChR expression and immunoregulatory function in peripheral blood monocytes and cognitive performance among 167 older adults (age 72.3 ± 7.6 years; 71% female). Penalized linear and logistic regression were used to identify nAChR-related features in classical, intermediate, and nonclassical monocytes, as well as immunophenotypes, clinical and sociodemographic factors, associated with cognitive status (Montreal Cognitive Assessment; MoCA). Intermediate monocytes had the highest expression of alpha-7 nAChRs and greater ex vivo inflammatory responses (83.7% TNF-ɑ^+^ cells) relative to classical (68.4%, *d* = 1.98, *P *< 0.001) or nonclassical monocytes (58.9%, *d* = 3.20, *P *< 0.001). Participants with mild cognitive impairment (MCI: *N* = 76) had higher soluble CD14 levels (1777 ± 377 pg/uL) and greater anticholinergic medication burden (ACB; mean = 1.76) than normocognitive participants (NC: *N* = 91; 1638 ± 352 pg/uL sCD14, *t*_155_ = 2.78, *P *= 0.006; mean ACB: 1.05, *t*_143_ = 3.13, *P *= 0.002). Multivariate regression models indicated that stronger nAChR-mediated immunoregulation in intermediate monocytes was associated with higher MoCA scores (beta = 0.13) and 14% lower odds of MCI, as well as lower ACB (beta = − 2.10; 95% CI − 4.14, − 0.61). This study demonstrates that peripheral monocytes exhibit subset-specific differences in nAChR phenotypes in older adults and provides preliminary evidence for their association with cognitive function and a potential mediating role between ACB and cognitive impairment.

**Supplementary Information:**

The online version contains supplementary material available at 10.1007/s10522-025-10220-3.

## Introduction

Nicotinic acetylcholine receptors (nAChRs) are important regulators of brain and immune function. nAChRs are expressed on neurons and glial cells in brain regions with fundamental roles in learning and memory, as well as on leukocytes where they modulate inflammation. During aging, changes in microglial function (e.g., loss of phagocytic capacity, decreased neurotrophic factor production) are thought to mediate neuropathology associated with Alzheimer’s disease and related dementias (ADRD) (Spittau 2017). In fact, multiple clinical trials using novel regulators of nAChRs, specifically the alpha-7 subtype (ɑ7nAChR), have been initiated for the treatment of ADRD due to their pro-cognitive and potentially disease-modifying effects (reviewed in Burns et al. 2023), though with mixed success. Bidirectional interactions between ɑ7nAChR and amyloid-beta (Aβ) by neurons and glial cells are thought to be integral for Aβ clearance and therefore involved in the pathophysiology of AD (Fontana et al. 2023). Indeed, loss of ɑ7nAChRs in post-mortem AD brain tissue may result from Aβ-nAChR complex formation and receptor internalization (Ren et al. 2020). Radiotracers to image ɑ7nAChRs in the human brain remain elusive, though preliminary work suggests reduced receptor binding in AD patients, as well as associations with Aβ accumulation and poorer cognitive performance (Nakaizumi et al. 2018). Conversely, greater ɑ7nAChR availability has been reported in mild cognitive impairment (MCI) (Coughlin et al. 2020), suggesting complex relationships between ɑ7nAChRs and ADRD status. Given these challenges, other biomarkers of ɑ7nAChR expression and activity, particularly in the periphery, could prove valuable in ascertaining ADRD risk or progression.

Preclinical studies indicate that the purported role of ɑ7nAChRs in Aβ clearance and deposition is partially mediated by their capacity to modulate inflammatory activity by brain astrocytes (Lykhmus et al. 2015, 2024). However, less is known about how their anti-inflammatory effects on peripheral immune cells are related to brain aging or ADRD vulnerability. Aging is accompanied by changes in the immune system that increase vulnerability to infection and the development of physical frailty, cardiovascular disease (CVD), and other chronic conditions, as well as increased mortality risk. One such change is chronic low-grade inflammation, or ‘inflammaging,’ which is associated with poorer age-related disease prognosis (e.g., dementia, frailty, CVD) and thought to be a lynchpin in biological aging. Multiple mechanisms for inflammaging have been proposed among both innate and adaptive immune cellular processes, such as increased NLRP3 inflammasome activation and impaired resolution mediators, and thus, modulating inflammation may be effective in the prevention of biological and cognitive aging.

Peripheral monocytes can infiltrate the brain under severe pathological conditions, but their role in AD-related processes within the CNS remains unclear (Muñoz-Castro et al. 2023). Irrespective of those that transmigrate into the brain, peripheral monocytes participate in Aβ clearance from blood and in inflammaging processes. For instance, peripheral monocytes derived from AD patients exhibit differential expression of Toll-like receptors 2 and 4 (TLR2/4), reduced phagocytosis of Aβ, and loss of anti-inflammatory functional pathways (e.g., IL-10) (see Bettcher et al. 2021) for review of peripheral-CNS crosstalk in AD). Interestingly, during AD progression, peripheral monocyte inflammatory activity may be highest at the mild cognitive impairment (MCI) phase (Munawara et al. 2021). Nevertheless, little is known about ɑ7nAChR-mediated immunomodulation by peripheral monocytes in aging. Peripheral monocytes can be divided into three major subsets (i.e., classical, intermediate, non-classical) based on cell surface maker expression, with proportional increases in intermediate and non-classical monocytes reported in AD (Thome et al. 2018); however, functional differences across subsets in ADRD remain uncharacterized. Furthermore, due to the higher prevalence of chronic conditions such as hypertension, older adults are often prescribed multiple pharmaceutical agents, many of which act as acetylcholine receptor antagonists (i.e., anticholinergic). Evidence suggests that 20–50% of older adults are prescribed anticholinergic drugs, which can have cumulative adverse effects, namely anticholinergic burden (ACB), including cognitive decline (Pieper et al. 2020). The mechanisms of ACB on cognition or causal links are unclear, but may involve immunomodulation (Sanghavi et al. 2022), possibly via ɑ7nAChR downregulation, desensitization, or functional alterations thereof.

The objective of the current study was to examine associations between cognitive test performance and cellular measures of nAChR-associated function in peripheral blood monocyte subsets while considering ACB among older adults living with a chronic condition of managed hypertension.

## Methods

### Study participants

This secondary analysis included 167 older adults (60–90 years) living with stage I and II hypertension (130 < SB*P *< 170 mmHg) who were recruited from the local community for a parent Tai Chi and healthy-aging intervention study (ClinicalTrials.gov: NCT02761603), regardless of antihypertensive medication use. Initial screening by telephone interview, followed by in-person confirmation, established the absence of the following exclusionary criteria: (1) inability to perform light to moderate exercise; (2) English-language illiteracy; (3) regular planned moderate exercise or meditation practice (≥ 2 × week and ≥ 30 min per episode); (4) cerebral neurological impairment, stroke, cardiac surgery or myocardial infarction within the past 12 months; (5) antipsychotic medication use, diagnosis of major depressive disorder by mental health professional within the past 6 months AND BDI-II score > 30, psychosis or substance-use disorder, suicidality; (6) autoimmune, inflammatory, or chronic infectious disorders, or health-related factors affecting immune function (e.g., current use of systemic immunomodulatory medication); (7) severe kidney disease; (8) inability to provide written informed consent; (9) oxygen-dependent chronic obstructive pulmonary disease; (10) current cancer treatment; or (11) BMI > 45 kg/m^2^.

### Clinical and anthropometric evaluations

The 30-item Montreal Cognitive Assessment (MoCA, version 7.1–7.3) was administered by study staff. Demographic variables and medical history were recorded via standardized interview, and medications were visually inspected and recorded from the label. Although hypertension was an inclusion criterion, blood pressure was pharmacologically well-controlled. Average basal systolic and diastolic blood pressure were calculated from three consecutive seated measurements at 5-min intervals following 15-min seated rest using an automated sphygmomanometer. Antihypertensive medications included beta-blockers, calcium-channel blockers, angiotensin-converting enzyme inhibitors, angiotensin-receptor blockers, and diuretics, and were summed to generate a score of antihypertensive medication burden. All medications were queried against a database of medications with known anticholinergic effects (Lozano-Ortega et al. 2020) and each participant’s corresponding ACB was summed based on the presence or absence of these medications and their respective ACB score. Chronic medical conditions, including coronary arterial disease, type-2 diabetes, chronic obstructive pulmonary disease, renal dysfunction, and cardiac arrythmia, were also summed based on presence/absence for each participant.

### Analysis of immune cell frequencies

Lymphocyte, monocyte, and neutrophil frequencies were quantified using complete blood count (CBC) with differential, performed by commercial laboratory (LabCorp, USA). Neutrophil counts were examined to screen participants for signs of active infection.

### Plasma immune markers of monocyte activation

Soluble CD14 (sCD14) concentrations (ug/L) were measured in duplicate using EDTA-treated plasma (1:200 dilution) using a commercial ELISA kit (Bio-Techne R&D Systems, Minneapolis, MN, USA; Cat. No. DC140) according to the manufacturer’s instructions. Plates were read on a VersaMax microplate reader (Molecular Devices, LLC, San Jose, CA, USA) and quantified using a five-parameter logistic curve. Concentration of plasma tumor necrosis factor alpha (TNF-ɑ) (ug/L) was measured using MSD assay kits (V-PLEX Proinflammation Panel 1) and the MSD Sector 2400 imager (MESO Scale Discovery). Samples were diluted 1:1000-fold and manufacturer-supplied lyophilized controls were used on each plate to confirm inter-assay variability. Intra-assay coefficients of variability (CV) were 0.71 and 9.89%, respectively.

### In vitro LPS-stimulation of monocytes

Fresh, heparinized whole peripheral blood (300uL) was loaded into flat-bottom, polystyrene 96-well plates and activated with 1 ug/mL of lipopolysaccharide (LPS; E.coli 0111:B4, Sigma-Aldrich, St. Louis, MO) for 30 min at 37C with 5% CO_2_. Paired samples from the same participants were stimulated with the selective ɑ7nAChR partial agonist, GTS-21 (Sigma-Aldrich, Cat #SML0326) at final concentrations of 0.25, 0.5, and 1.0 mM in addition to LPS. Additional replicates were stimulated with (-)-nicotine (Sigma-Aldrich, Cat #SML1236) at final concentrations of 0.5, 1.25, and 2.50 mM in addition to LPS. These concentrations were empirically determined to minimize cell death (< 5%) and provide a replicable dose–response suppression of intracellular TNF-ɑ expression when co-administered with LPS (see Supplemental Fig. 1). To inhibit cytokine excretion, thus allowing for intracellular detection of TNF-ɑ, Brefeldin A (10 ug/mL) was added to each sample for the final 3 h of incubation.

**Fig. 1:**
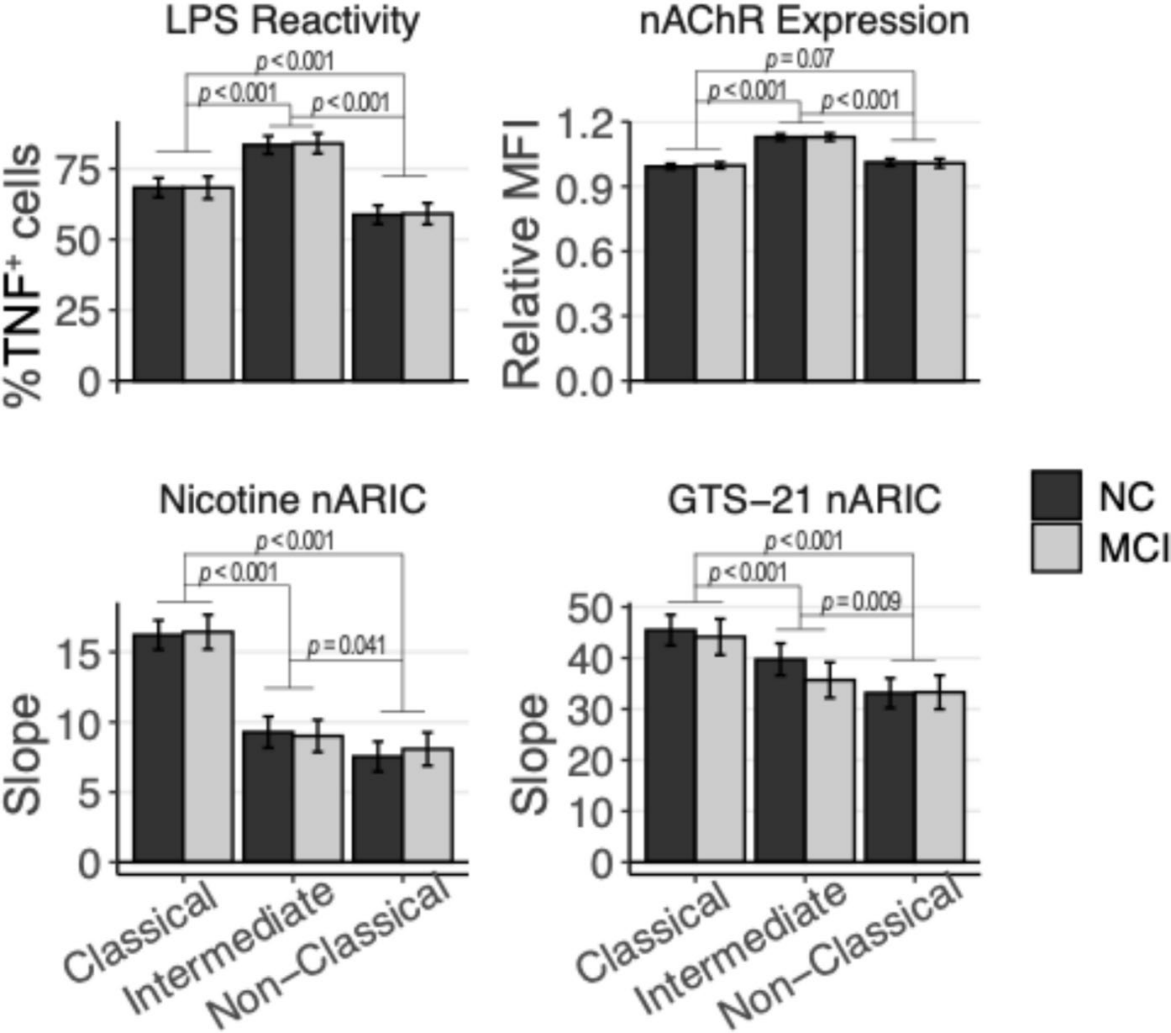
Peripheral monocyte subset phenotypes in older adults with normocognitive (NC) or mild cognitive impairment (MCI) status. Monocyte subsets are indicated on the x-axis and were discerned through flow cytometry (see Sect. “Flow cytometry”) as classical (DR^+^CD14^+^CD16^−^), intermediate (DR^+^CD14^+^CD16^+^), or nonclassical (DR^+^CD14^dim^CD16^+^) cells. Alpha-7 nicotinic acetylcholine receptor (nAChR) expression based on relative median fluorescence intensity (MFI), normalized within-subjects to their respective MFI derived from all monocytes. Immunoregulation (nARIC) values reflect monocyte subset sensitivity to suppression of intracellular TNF-ɑ (as determined by flow cytometry) in response to 1 ug/mL lipopolysaccharide (LPS) and 3.5 h co-incubation with varying doses of nAChR agonist (GTS-21 or nicotine). Higher slope (y-axis) values denote stronger immunoregulation (arbitrary units). LPS reactivity was defined as the proportion of %TNF-ɑ^+^ monocytes within each subset after 3.5 h incubation with LPS (see Sect. “In vitro LPS-stimulation of monocytes”). Error bars indicate 95% confidence intervals. *P*-values derived from Tukey-adjusted *t*-tests

### Flow cytometry

#### Quantification of monocyte subset proportions

Monocyte subsets were analyzed using 300uL of fresh whole blood, collected into heparin-treated tubes. For each sample, data from 10,000 cells were acquired and analyzed using a FACScalibur flow cytometer and FlowJo software (v10, TreeStar, Ashland, OR). Gating strategy is shown in Supplemental Fig. 1. Briefly, forward (FSC) and side (SSC) scatters were used to gate monocytes and to exclude cellular debris and doublets. An electronic gate was placed on HLA-DR^+^ cells, which were then gated on CD14^+^ and CD16^+^. Classical monocytes were defined as HLA-DR^+^CD14^+^CD16^−^, intermediate monocytes as HLA-DR^+^CD14^+^CD16^+^, and nonclassical monocytes as HLA-DR^+^CD14^dim^CD16^+^ (i.e., three subset gates). A fourth gate was created that included all three subset gates (i.e., one all monocyte gate) and subset proportions were calculated for each participant as the ratio of that subset to all monocytes.

#### nAChR-mediated inflammation control (nARIC)

Quantification of intracellular TNF-ɑ within each monocyte subset using fluorochrome-conjugated anti-TNF-ɑ staining was performed as previously described (Kohn et al. 2019), following staining and fixation of cell surface markers. Incubation concentrations of nicotine (0.5, 1.25, and 2.50 mM) and GTS-21 (0.25, 0.50, and 1.00 mM) were empirically determined to minimize cell death and achieve quasi-linear dose–response curves (including a 0 mM control condition). To quantify participant-specific nAChR function, separate linear mixed-effects models were implemented, with random slope-intercept, to fit linear dose–response curves for nicotine and GTS-21 to %TNF-ɑ^+^ cells within each monocyte subset (Supplemental Fig. 2). Thus, for each participant, slope values were derived for (i) classical; (ii) intermediate; and (iii) non-classical monocytes for nicotine and GTS-21 (i.e., 6 values per participant). Steeper slopes (i.e., more negative values) indicate greater suppression of TNF-ɑ expression in response to nAChR agonist and therefore greater inflammation control, or ‘nARIC.’ To aid interpretation, values were multiplied by − 1 such that higher values indicate greater inflammation control.

#### Monocyte ɑ7nAChR relative expression

Quantification of ɑ7nAChR expression on monocyte subsets was performed by co-staining with the competitive agonist, alpha-bungarotoxin-FITC, which binds irreversibly to the ɑ subunit of nAChRs with high affinity. After gating monocyte subsets (Sect. “Quantification of monocyte subset proportions”), geometric mean fluorescence intensity (MFI) values were extracted within each subset gate and the all-monocyte gate. PMT voltages were periodically optimized across experiments; therefore, to make inter-individual comparisons and inferences, subset-specific MFI values were normalized within-participants by dividing by each subset’s MFI value by the MFI value for all monocytes.

### Statistical analysis

All analyses were conducted in *R* using RStudio software. Data were visually inspected for outliers, and values ± 4 SD from the sample mean were excluded. Descriptive statistics were computed in initial analyses, and cognitive status subgroups were compared using independent samples *t*-tests and Chi-square tests for continuous and categorical variables, respectively. Data missingness ranged 1–10% for variables unrelated to the cellular assays (MoCA scores were available for all patients), and 17–42% for those derived from cellular assays. For the latter, missingness was attributable to blood collection difficulties and technical issues, as all cellular assays (e.g., incubation, cell staining, flow cytometry acquisition) were performed same-day requiring freshly-drawn blood samples. Notably, %TNF-ɑ^+^ data for at least 1 of the 4 analyte concentrations used to compute nARIC were available for 83% of participants. Missing data were multiply imputed (*m* = 20, maxit = 10) under the missing at random mechanism using unsupervised random forest-based imputation in *mice*. Each participant’s nARIC values were derived within each imputed dataset. Linear mixed-effects models with robust scoring equations (Koller and Stahel 2023) were used to evaluate associations between ɑ7nAChR expression, LPS-induced TNF-ɑ^+^ cell proportions, and nARIC values, with an intercept-only random effect of participant on each imputed dataset. Monocyte subset-specific effects were interrogated by including subset type as a moderator (i.e., interaction term). Point and 95% confidence interval estimates were pooled across the multiply imputed data according to Rubin’s rules. Conditional means and post-hoc contrasts were computed and pooled, with Tukey-adjusted *p*-values reported.

To identify cellular, sociodemographic, and other clinical variables associated with MCI and MoCA scores, regularized linear and logistic regression models (i.e., ridge regression) were trained on each imputed dataset (80:20 split for training:testing) with ten-fold cross-validation for hyperparameter tuning (e.g., lambda) based on minimization of mean squared error and deviance, respectively. A total of 24 features were normalized and filtered to the top 10 features (based on mean decrease in gini impurity; *ranger* filter) within each training set to avoid overfitting and *P *>  > *n* (e.g., more than 10 observations per feature). Due to the random nature of train-test splitting and inconsistently filtered and fit features selected across datasets, only for variables with an inclusion frequency ≥ 50% across models were coefficients determined by pooling estimates and performance metrics based on prediction on the test set (e.g., *R*^2^, c-statistic), defined as the mean across all models (Gunn et al. 2023). This inclusion frequency threshold has been shown to balance the bias-variance trade-off for applied and simulation studies (Lachenbruch 2011).

## Results

### Participant characteristics

Sociodemographic and clinical characteristics of the study population are summarized in Table 1, grouped into normocognitive (54.5% NC; MoCA > 25) or mild cognitive impairment (45.5% MCI; MoCA ≤ 25). Participants with MCI were ~ 4 years older (*t*_159_ = 2.94, *P *= 0.003), prescribed more antihypertensive medications (*t*_143_ = 2.47, *P *= 0.015), and presented with greater ACB (*t*_143_ = 3.13, *P *= 0.002). Participants in the MCI group also had higher levels of circulating sCD14 in plasma than those in the NC group (*t*_155_ = 2.78, *P *= 0.006).

**Table 1:** Sociodemographic and clinical characteristics of study population, grouped into normocognitive (NC; MoCA > 25) or mild cognitive impairment (MCI; MoCA ≤ 25) status

Variable	All participants	Normo-cognitive (NC)	Mild cognitive impairment (MCI)
*N*	167	91	76
Age (years)	72.3 (7.6)	**70.7 (7.2)**	**74.3 (7.7)**
% Female	70.7%	65.9%	76.3%
Race (%White)	84.4%	86.8%	81.6%
% Married/Partnered	34.1%	35.2%	33.3%
% College educated	54.5%	61.5%	46.1%
SBP (mmHg)	134.8 (18.3)	132.9 (16.3)	137.2 (20.3)
DBP (mmHg)	69.4 (9.8)	70.4 (9.3)	68.1 (10.2)
Other chronic medical conditions (*N*)	0.83 (0.57)	0.79 (0.50)	0.89 (0.65)
Antihypertensive medications (*N*)	1.26 (1.1)	**1.04 (0.85)**	**1.51 (1.3)**
BMI (kg/m^2^)	29.4 (6.5)	29.6 (6.4)	29.0 (6.6)
MoCA score	25.3 (3.4)	**27.7 (1.4)**	**22.7 (2.7)**
Monocyte count (cells/uL)	482 (166)	475 (162)	491 (164)
BDI-II score	6.86 (6.7)	6.81 (5.5)	6.91 (6.0)
Participants taking medications with ACB (%)	60.5%	53.8%	68.4%
Mean ACB^*^ Score	1.41 (1.2)	**1.05 (1.0)**	**1.76 (1.3)**
Smoking history (% yes)	33.5%	36.3%	30.3%
% Classical monocytes (DR^+^CD14^+^CD16^−^)	76.6 (8.5)	77.3 (6.3)	75.7 (7.2)
% Intermediate monocytes (DR^+^CD14^+^CD16^+^)	5.2 (2.2)	4.8 (1.3)	5.5 (2.0)
% Nonclassical monocytes (DR^+^CD14^dim^CD16^+^)	17.5 (6.5)	17.2 (5.5)	17.8 (5.1)
Plasma sCD14 (pg/uL)	1698 (370)	**1638 (352)**	**1777 (377)**
Plasma TNF-ɑ (pg/mL)	1.32 (0.61)	1.31 (0.69)	1.33 (0.48)

Monocyte subset proportions are relative to all monocytes, as defined in Sect. “Quantification of monocyte subset proportions”. BMI = body mass index (kg/m^2^); ACB = anticholinergic burden; BDI-II = Beck Depression Inventory; MoCA = Montreal Cognitive Assessment; SB*P *= systolic blood pressure; SB*P *= diastolic blood pressure; MCI = mild cognitive impairment. ‘Antihypertensive medications (N)’ reflects average number of unique drugs per participant

*Mean ACB score derived from participants (*N* = 88) taking at least 1 medication with non-zero ACB score. Bold typeface indicates significant difference between normocognitive (NC) and mild cognitive impairment (MCI) groups at *P *< 0.05, uncorrected, based on multiply imputed, pooled, chi-square or independent samples *t*-tests for categorical and continuous variables, respectively

### alpha7-nAChR expression and immunoregulation in peripheral blood monocytes

Across all participants, intermediate monocytes had significantly higher relative expression of ɑ7nAChRs compared to classical (13.4% difference, *t* = 17.9, *P *< 0.001) and non-classical (11.9% difference, *t* = 13.7, *P *< 0.001) monocytes (Fig. 1). Expression levels did not differ between classical and non-classical monocytes (1.5% difference, *t* = 1.84, *P *= 0.07). Acute inflammatory reactivity, as measured by LPS-evoked TNF-ɑ^+^ cell proportions, were also higher in intermediate monocytes (83.7%, 95% CI 81.3, 86.0) relative to classical (68.4%, 95% CI 65.7, 71.0; *d* = 1.98, *t* = 15.7, *P *< 0.001) and nonclassical monocytes (58.9%, 95% CI 56.4, 61.5; *d* = 3.20, *t* = 25.9, *P *< 0.001). Classical monocytes had a larger proportion of TNF-ɑ^+^ cells in response to LPS stimulation than non-classical monocytes (*d* = 1.22, *t* = 9.41, *P *< 0.001).

Immunoregulation of TNF-ɑ production by nAChR agonists was quantified in each monocyte subset. Immunoregulation by each agonist (nicotine and GTS-21) was moderately correlated within classical (*r* = 0.67, 95% CI 0.53, 0.78; *P *< 0.001) and nonclassical subsets (*r* = 0.58, 95% CI 0.41, 0.71; *P *< 0.001), and weakly correlated within intermediate monocytes (*r* = 0.22, *P *= 0.08). Classical monocytes exhibited greater immunoregulation than intermediate (nicotine: *β* = 7.17, 95% CI 5.93, 8.41; *t* = 11.4, *P *< 0.001; GTS-21: *β* = 6.99, 95% CI 3.76, 10.2; *t* = 4.25, *P *< 0.001) and nonclassical monocytes (nicotine: *β* = 8.53, 95% CI 7.39, 9.67; *t* = 14.8, *P *< 0.001; GTS-21: *β* = 11.7, 95% CI 8.55, 14.8; *t* = 7.38, *P *< 0.001), and nonclassical monocytes were less sensitive to agonist than intermediate monocytes (nicotine: *β* = 1.36, 95% CI 0.26, 2.47; *t* = 2.42, *P *= 0.041; GTS-21: *β* = 4.66, 95% CI 1.56, 7.77; *t* = 2.95, *P *= 0.009). Immunoregulation within classical and non-classical monocytes was positively correlated for nicotine (*r* = 0.28, 95% CI 0.04, 0.48; *P *= 0.02) and GTS-21 (*r* = 0.58, 95% CI 0.44, 0.70; *P *< 0.001). Contrary to expectation, higher ɑ7nAChR expression was not associated with immunomodulation by nAChR agonists within any monocyte subset.

### Associations with cognitive status and clinical characteristics

**Fig. 2:**
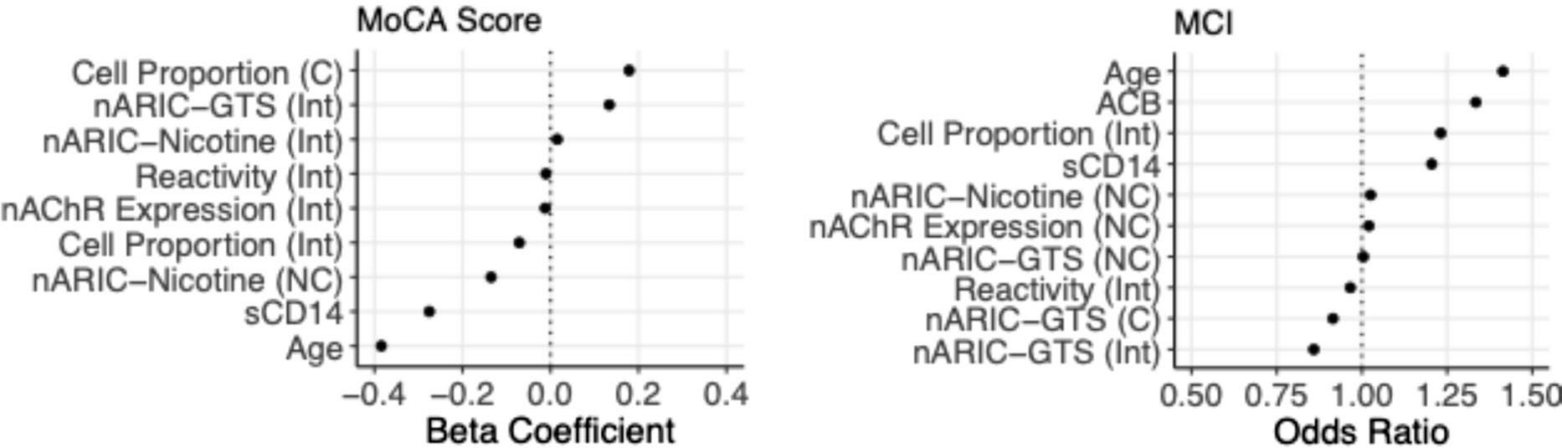
Pooled, standardized beta coefficients and odds ratios for predictors derived from penalized linear (left panel) and logistic (right panel) regression models testing associations with MoCA scores and MCI (vs. normocognitive) status, respectively. Models were trained on 80% of the original data and tested on the residual 20% set. Monocyte subsets indicated in parentheses, where applicable (C: classical, NC: non-classical, Int: intermediate). Beta coefficients > 0 indicate positive associations with MoCA score (i.e., better cognitive performance), and odds ratios > 1 indicate positive associations with MCI status (i.e., greater odds of scoring < 26 points on MoCA)

Penalized regression (mean *R*^*2*^ = 7.4%) and classification (c-statistic = 0.68) models were implemented to identify monocyte phenotypes, clinical, and sociodemographic features associated with cognitive status. As expected, older age was the strongest predictor of worse MoCA scores (beta = − 0.38) and MCI status (OR 1.41) (Fig. 2). For the regression model, lower MoCA scores (i.e., poorer cognitive status) were associated with higher plasma soluble CD14 (b = − 0.28), stronger immunoregulation within nonclassical monocytes (b = − 0.14), and higher intermediate monocyte proportions (b = − 0.07) **(**Fig. 2). Higher MoCA scores were associated with greater classical monocyte proportions (b = 0.18) and stronger immunoregulation within intermediate monocytes (b = 0.13). For the classification model, greater odds of MCI status were associated with higher acetylcholinergic medication burden (OR 1.33), greater intermediate monocyte proportions (OR 1.23), and higher soluble CD14 levels (OR 1.20). Stronger immunoregulation by intermediate monocytes was associated with lower odds of MCI (OR 0.86).

### Acetylcholinergic medication burden

Given the observed association between MCI/MoCA scores and monocyte phenotypes, as well as associations between ACB and MCI status, we tested whether ACB scores were also related to monocyte phenotypes. Greater ACB was associated with higher acute inflammatory reactivity (b = 2.52, 95% CI 0.47, 4.57), although not in a monocyte subset-specific manner. Expression of ɑ7nAChRs across monocyte subsets was not associated with ACB; however, higher ACB was associated with poorer immunoregulation by GTS-21 in intermediate monocytes (b = − 2.10; − 4.14, − 0.61), but not in classical or nonclassical monocytes. As expected, participants with more medical comorbidities had higher ACB (b = 0.11; 0.05, 0.18).

## Discussion

In this investigation of the relationship between cholinergic system-mediated regulation of inflammatory responses in peripheral monocytes and aging-related cognitive decline, we identified previously unreported functional differences between monocyte subsets. Specifically, intermediate monocytes expressed significantly higher density of ɑ7nAChR than classical or non-classical subsets, which was accompanied by a more pronounced inflammatory response to LPS challenge, measured by intracellular TNF-ɑ production. In addition, we implemented a novel ex vivo assay to evaluate nAChR-mediated inflammation control in monocytes and identified subset-specific differences, such that classical monocytes were the most sensitive to anti-inflammatory effects of nAChR agonist, followed by intermediate and non-classical monocytes. Sensitivity was correlated between agonists in classical and non-classical monocytes (i.e., more sensitivity to nicotine was associated with more sensitivity to GTS-21), but only weakly in intermediate cells, suggesting somewhat distinct signal transduction mechanisms between nicotine and GTS-21 agonism of nAChRs within intermediate monocytes. Unexpectedly, greater ɑ7nAChR expression was not associated with stronger immunoregulation by the receptor agonists, which suggests that factors beyond expression alone mediated receptor function. While ɑ7-mediated activation is understood to be the canonical pathway by which nAChRs exert anti-inflammatory effects, ɑ7-independent processes are believed to influence their immunomodulatory capacity (Garg and Loring 2019). Other monocyte inflammatory markers induced by LPS, such as interleukin-(IL)-6 or upstream signaling molecules, may yield additional insights to nAChR immunomodulatory function, but were not examined in our analysis.

Multivariate regression identified several predictors of global cognitive function. Notably, higher sCD14 levels were associated with poorer cognitive status, which has been previously reported in large epidemiological cohort studies (Pase et al. 2020). In addition, higher proportions of classical and lower proportions of intermediate monocytes were associated with better cognition, which has been previously reported to correlate with younger age (Cao et al. 2022). Also in alignment with this finding, greater intermediate monocyte proportions are reportedly associated with lower global neuropsychological and executive function in women living with HIV (Veenhuis et al. 2021).

Our results also provide preliminary evidence that monocyte subset sensitivity to nAChR-mediated immunoregulation is associated with cognitive status. Specifically, decreased sensitivity of intermediate monocytes to GTS-21 was independently associated with poorer cognition, but not their inflammatory reactivity or their expression of ɑ7nAChRs. While multiple mechanisms may mediate this relationship, we also found that reduced intermediate monocyte immunoregulation was associated with higher ACB, and that higher ACB was independently predictive of MCI. While the analytical sample was underpowered to formally test whether the relationship between ACB and MCI was mediated by lower immunoregulation in intermediate monocytes, these data provide initial clues pointing to dysregulated acetylcholinergic signaling in intermediate blood monocytes as a potential mediator and/or peripheral biomarker of ACB-associated cognitive dysfunction. Given the role of nAChRs, specifically those with the ɑ7 subunit, in Aβ metabolism (Roberts et al. 2021), future studies of monocyte nAChR function, ACB, and cognition should concurrently quantify Aβ 42/40 ratios in peripheral blood/CSF or brain Aβ levels using neuroimaging to evaluate their associations with validated neuropathological biomarkers.

The present study had several limitations. First, participants were not classified as MCI based on a comprehensive neuropsychological examination, rather using a global cognitive screening tool (i.e., MoCA). While MoCA has higher sensitivity and specificity for MCI detection compared to the Mini-Mental State Examination (MMSE) (Trzepacz et al. 2015), ceiling effects in well-educated populations may exist, though only 11 (6.6%) of 167 participants scored 30/30 in our sample; however, it remains possible that some participants were misclassified. Future studies would benefit from implementing a neuropsychological battery, which would also permit detection of domain-specific changes in cognition (e.g., working memory, executive function) that may be associated with peripheral monocyte phenotypes or ACB, but cannot be interrogated using a global instrument. Nevertheless, graded associations between total MoCA score and the outcomes of interest were examined here, in addition to threshold score-based classification, which yielded similar findings. Second, medication-related ACB as quantified in this study may be a proxy for general health status rather than ACB per se. In other words, individuals with more chronic illnesses and medical comorbidities will simply have greater polypharmacy and therefore ACB. However, our multivariate analyses adjusted for anti-hypertensive medication burden and the total number of chronic medical conditions, and the association between ACB and MCI persisted. Third, additional unexamined mechanisms beyond ɑ7nAChR expression may mediate the anti-inflammatory effects of acetylcholine agonists ex vivo. Fourth, our monocyte phenotyping protocol was limited to surface expression of CD14 and CD16, whereas age-related functional markers of activation and adhesion (e.g., CD88, CD11b) may reveal more nuanced relationships with cognitive function that were undetected here. Additionally, our study population was restricted to older adults, though future studies should explore and test for age-related changes in ɑ7nAChR expression and function in peripheral monocytes, which may offer additional insights into mechanisms of accelerated cognitive and biological aging in middle age. Lastly, the small size and socio-demographically distinct study sample (e.g., majority college educated, ~ 85% White) may limit the generalizability of the findings to more diverse individuals or to the broader population.

In summary, our study demonstrates that peripheral blood monocytes exhibit subset-specific differences in nAChR-mediated anti-inflammatory function among older adults. Furthermore, our analysis provides preliminary evidence that monocyte phenotypes are related to global cognitive function and may mediate associations between greater anticholinergic medication burden and cognitive impairment, though larger studies are needed to systematically test this hypothesis. The immunomodulatory role of the cholinergic system in CNS outcomes (Ramos-Martínez et al. 2021) underscores the importance of further elucidating nAChR-mediated modulation of cellular inflammatory activity in brain aging and cognition and the major cellular and molecular players involved.

## Supplementary Information

Below is the link to the electronic supplementary material.

Supplementary file1 (DOCX 283 KB)

## Data Availability

The code used for the analysis and the underlying deidentified data are available from the corresponding author upon reasonable request.

## References

[CR1] Bettcher BM, Tansey MG, Dorothée G, Heneka MT (2021) Peripheral and central immune system crosstalk in Alzheimer disease—a research prospectus. Nat Rev Neurol 17:689–701. 10.1038/s41582-021-00549-x 34522039 10.1038/s41582-021-00549-x PMC8439173

[CR2] Burns LH, Pei Z, Wang H (2023) Targeting α7 nicotinic acetylcholine receptors and their protein interactions in Alzheimer’s disease drug development. Drug Dev Res 84:1085–1095. 10.1002/ddr.22085 37291958 10.1002/ddr.22085

[CR3] Cao Y, Fan Y, Li F et al (2022) Phenotypic and functional alterations of monocyte subsets with aging. Immun Ageing 19:63. 10.1186/s12979-022-00321-9 36514074 10.1186/s12979-022-00321-9 PMC9745938

[CR4] Coughlin JM, Rubin LH, Du Y et al (2020) High availability of the α7-nicotinic acetylcholine receptor in brains of Individuals with mild cognitive impairment: a pilot study using^18^ F-ASEM PET. J Nucl Med 61:423–426. 10.2967/jnumed.119.230979 31420499 10.2967/jnumed.119.230979 PMC9374031

[CR5] Fontana IC, Kumar A, Nordberg A (2023) The role of astrocytic α7 nicotinic acetylcholine receptors in Alzheimer disease. Nat Rev Neurol 19:278–288. 10.1038/s41582-023-00792-4 36977843 10.1038/s41582-023-00792-4

[CR6] Garg BK, Loring RH (2019) GTS-21 has cell-specific anti-inflammatory effects independent of α7 nicotinic acetylcholine receptors. PLoS ONE 14:e0214942. 10.1371/journal.pone.0214942 30947238 10.1371/journal.pone.0214942 PMC6448884

[CR7] Gunn HJ, Hayati Rezvan P, Fernández MI, Comulada WS (2023) How to apply variable selection machine learning algorithms with multiply imputed data: a missing discussion. Psychol Methods 28:452–471. 10.1037/met0000478 35113633 10.1037/met0000478 PMC10117422

[CR8] Kohn JN, Cabrera Y, Dimitrov S et al (2019) Sex-specific roles of cellular inflammation and cardiometabolism in obesity-associated depressive symptomatology. Int J Obes 43:2045–2056. 10.1038/s41366-019-0375-3 10.1038/s41366-019-0375-3 PMC6774832 31089263

[CR9] Koller M, Stahel WA (2023) Robust estimation of general linear mixed effects models. In: Yi M, Nordhausen K (eds) Robust and multivariate statistical methods. Springer International Publishing, Cham, pp 297–322

[CR10] Lachenbruch PA (2011) Variable selection when missing values are present: a case study. Stat Methods Med Res 20:429–444. 10.1177/0962280209358003 20442196 10.1177/0962280209358003

[CR11] Lozano-Ortega G, Johnston KM, Cheung A et al (2020) A review of published anticholinergic scales and measures and their applicability in database analyses. Arch Gerontol Geriatr 87:103885. 10.1016/j.archger.2019.05.010 31155228 10.1016/j.archger.2019.05.010

[CR12] Lykhmus O, Voytenko L, Koval L et al (2015) α7 nicotinic acetylcholine receptor-specific antibody induces inflammation and amyloid β42 accumulation in the mouse brain to impair memory. PLoS ONE 10:e0122706. 10.1371/journal.pone.0122706 25816313 10.1371/journal.pone.0122706 PMC4376778

[CR13] Lykhmus O, Tzeng W-Y, Koval L et al (2024) Impairment of brain function in a mouse model of Alzheimer’s disease during the pre-depositing phase: the role of α7 nicotinic acetylcholine receptors. Biomed Pharmacother 178:117255. 10.1016/j.biopha.2024.117255 39116785 10.1016/j.biopha.2024.117255

[CR14] Munawara U, Catanzaro M, Xu W et al (2021) Hyperactivation of monocytes and macrophages in MCI patients contributes to the progression of Alzheimer’s disease. Immun Ageing 18:29. 10.1186/s12979-021-00236-x 34154615 10.1186/s12979-021-00236-x PMC8215492

[CR15] Muñoz-Castro C, Mejias-Ortega M, Sanchez-Mejias E et al (2023) Monocyte-derived cells invade brain parenchyma and amyloid plaques in human Alzheimer’s disease hippocampus. Acta Neuropathol Commun 11:31. 10.1186/s40478-023-01530-z 36855152 10.1186/s40478-023-01530-z PMC9976401

[CR16] Nakaizumi K, Ouchi Y, Terada T et al (2018) In vivo depiction of α7 nicotinic receptor loss for cognitive decline in Alzheimer’s disease. J Alzheimer’s Dis 61:1355–1365. 10.3233/JAD-170591 29376856 10.3233/JAD-170591

[CR17] Pase MP, Himali JJ, Beiser AS et al (2020) Association of CD14 with incident dementia and markers of brain aging and injury. Neurology. 10.1212/WNL.0000000000008682 31818907 10.1212/WNL.0000000000008682 PMC7108812

[CR18] Pieper NT, Grossi CM, Chan W-Y et al (2020) Anticholinergic drugs and incident dementia, mild cognitive impairment and cognitive decline: a meta-analysis. Age Ageing 49:939–947. 10.1093/ageing/afaa090 32603415 10.1093/ageing/afaa090 PMC7583519

[CR19] Ramos-Martínez IE, Rodríguez MC, Cerbón M et al (2021) Role of the cholinergic anti-inflammatory reflex in central nervous system diseases. IJMS 22:13427. 10.3390/ijms222413427 34948222 10.3390/ijms222413427 PMC8705572

[CR20] Ren J-M, Zhang S-L, Wang X-L et al (2020) Expression levels of the α7 nicotinic acetylcholine receptor in the brains of patients with Alzheimer’s disease and their effect on synaptic proteins in SH-SY5Y cells. Mol Med Rep 22:2063–2075. 10.3892/mmr.2020.11253 32582986 10.3892/mmr.2020.11253 PMC7411404

[CR21] Roberts JP, Stokoe SA, Sathler MF et al (2021) Selective coactivation of α7- and α4β2-nicotinic acetylcholine receptors reverses beta-amyloid–induced synaptic dysfunction. J Biol Chem 296:100402. 10.1016/j.jbc.2021.100402 33571523 10.1016/j.jbc.2021.100402 PMC7961090

[CR22] Sanghavi R, Pana TA, Mamayusupova H et al (2022) Higher anticholinergic burden from medications is associated with significant increase in markers of inflammation in the EPIC-Norfolk prospective population-based cohort study. Br J Clin Pharma 88:3297–3306. 10.1111/bcp.15261 10.1111/bcp.15261 PMC9373850 35118716

[CR23] Spittau B (2017) Aging microglia—phenotypes, functions and implications for age-related neurodegenerative diseases. Front Aging Neurosci 9:194. 10.3389/fnagi.2017.00194 28659790 10.3389/fnagi.2017.00194 PMC5469878

[CR24] Thome AD, Faridar A, Beers DR et al (2018) Functional alterations of myeloid cells during the course of Alzheimer’s disease. Mol Neurodegeneration 13:61. 10.1186/s13024-018-0293-1 10.1186/s13024-018-0293-1 PMC6233576 30424785

[CR25] Trzepacz PT, Wang S, Walker B, Saykin AJ (2015) Relationship between the Montreal cognitive assessment and mini-mental state examination for assessment of mild cognitive impairment in older adults. BMC Geriatr 15:107. 10.1186/s12877-015-0103-3 26346644 10.1186/s12877-015-0103-3 PMC4562190

[CR26] Veenhuis RT, Williams DW, Shirk EN et al (2021) Higher circulating intermediate monocytes are associated with cognitive function in women with HIV. JCI Insight. 10.1172/jci.insight.146215 33914710 10.1172/jci.insight.146215 PMC8262276

