# Supplementary material for: Elucidating the role of peripheral monocyte nicotinic acetylcholine receptors and inflammation in cognitive outcomes in older adults
Source: Biogerontology. 2025 Mar 30;26(2):82. doi: 10.1007/s10522-025-10220-3 (PMC11955431; doi:10.1007/s10522-025-10220-3)
Supplement: Supplementary file 1 — Supplementary file1 (DOCX 283 KB) [file 10522_2025_10220_MOESM1_ESM.docx]

**SUPPLEMENTARY INFORMATION**

**Supplemental Fig. 1.** Flow cytometry gating strategy. (right panels) Monocyte subsets were identified from unstimulated whole blood by FSC/SSC gating, HLA-DR positivity, and expression of CD14 and CD16 as follows: classical monocytes were defined as HLA-DR^+^CD16^-^CD14^+^, intermediate monocytes as HLA-DR^+^CD16^+^CD14^+^, and nonclassical monocytes as HLA-DR^+^CD16^+^CD14^dim^. Quantification of ɑ7 nicotinic acetylcholine receptor (ɑ7nAChR) expression was determined by median fluorescence intensity (MFI) of anti-bungarotoxin (BTX) within each monocyte subset. Raw MFI values were normalized within-subjects by dividing each subset’s MFI by the MFI value of “all monocytes,” which were an aggregation of all three subset gates. (left panels) Nicotinic acetylcholine receptor-mediated inflammation control (nARIC) was quantified by LPS stimulation and co-incubation with nAChR agonists (nicotine and GTS-21, separately), followed by intracellular staining for TNF-ɑ and gating TNF-ɑ-expressing subsets. Note that due to loss of CD16 expression by monocytes following stimulation, subsets were identified by HLA-DR expression for nARIC quantification, which has been previously shown to serve as a reliable proxy for CD16 in monocyte subset identification (Dimitrov et al. 2013).

**Supplemental Fig. 2.** %TNF-ɑ^+^ classical monocytes (HLA-DR^+^CD16^-^CD14^+^ cells) and individual-specific slopes derived from LMMs for nARIC computation. Intracellular TNF-ɑ expression was quantified using FITC-anti-TNF-ɑ staining, performed as previously described (Kohn et al. 2019), following staining and fixation of cell surface markers. To quantify participant-specific slopes, a linear mixed-effects model was implemented, with random slope-intercept, to fit linear dose-response curves. Greater suppression of %TNF-ɑ^+^ cells yielded steeper (i.e., more negative) slopes. To aid interpretation, values were multiplied by -1 such that higher values indicate greater suppression (i.e., more inflammation control, or ‘nARIC’) in downstream analyses.
